# The use of robotic upper limb therapy in routine clinical practice for stroke survivors: Insights from Australian therapists

**DOI:** 10.1111/1440-1630.70010

**Published:** 2025-03-25

**Authors:** Nicholas Flynn, Elspeth Froude, Deirdre Cooke, Suzanne Kuys

**Affiliations:** ^1^ Australian Catholic University Brisbane Queensland Australia

**Keywords:** acceptance, occupational therapy, physical therapy, robotics, stroke rehabilitation, upper extremity

## Abstract

**Introduction:**

There is a limited understanding of therapist acceptance and use of robot‐assisted upper limb therapy (RT‐ULT) in routine practice. The aim of this study was to explore the factors that influence Australian therapist acceptance and use of RT‐ULT.

**Methods:**

Two discipline‐specific focus groups were conducted involving occupational therapists (*n* = 5) and physiotherapists (*n* = 4) who had used RT‐ULT. Focus group questions were developed, and transcriptions analysed using the Theoretical Domains Framework (TDF). Additionally, participants scored the overall usability of the RT‐ULT device with the System Usability Scale (SUS).

**Consumer and Community Involvement:**

There was no direct involvement from consumers or community in this study.

**Findings:**

Nine of the 14 domains of the TDF were covered in depth by participants during the focus groups: environmental context and resources, beliefs about consequences, knowledge, skills, decision‐making, reinforcement, social influences, social/professional role and identity (single domain), and beliefs about capabilities. Physiotherapists recorded higher scores of the device on the SUS than the occupational therapists.

**Conclusion:**

Both disciplines were accepting of RT‐ULT, but it was physiotherapists who predominantly used RT‐ULT in part due to the device being located in the physiotherapy rehabilitation gym. Other factors facilitating RT‐ULT acceptance in practice included (1) increase in repetitive, intensive independent practice for stroke survivors, (2) ease of use, (3) strong patient acceptance, and (4) implementation process being clinician‐led. Functional‐based UL practice took priority over RT‐ULT once stroke survivors demonstrated sufficient active movement and RT‐ULT was not used in isolation but part of a combination of UL interventions.

**PLAIN LANGUAGE SUMMARY:**

There is a little known about what therapists think about using robot‐assisted upper limb therapy in their daily practice. The aim of this study was to explore Australian therapist perceptions of the use of robotics.

Focus groups were conducted separately with five occupational therapists and four physiotherapists who had used robotics at their rehabilitation facility. In addition to the focus groups, each therapist scored the user‐friendliness of the robotic device by completing a short survey.

Both occupational therapists and physiotherapists believed the robotics was a beneficial addition to the rehabilitation facility. Physiotherapists used the device more than the occupational therapists with the device being located in the physiotherapy area of the rehabilitation facility. Therapists explained that robotics increased the amount of practice stroke survivors could do, was easy to use, and was motivating for stroke survivors. However, once stroke survivors had gained enough arm movement, the focus moved to practicing actual daily tasks rather than robotics. Also, robotics was not the only form of upper limb therapy offered to stroke survivors with multiple upper limb treatments used to aid their recovery.

**Key Points for Occupational Therapy**

- Therapist acceptance and usability of the robotic device were supported by the simple functionality of the device, motivation for patients, and capacity to enable semi‐supervised repetitive intensive practice.
- Device location, clinical priorities, and staff employment arrangements also influenced the use of the upper limb robotic device.
- Robotics was just one of a range of upper limb interventions provided to patients and used primarily in the early phase of patient recovery.

## 1 INTRODUCTION

There is a need to continue to develop and implement interventions that are effective in facilitating upper limb (UL) recovery for stroke survivors (Hayward et al., 2021; Stinear et al., 2020). Robot‐assisted upper limb therapy (RT‐ULT) has emerged in recent years with the potential to improve UL outcomes for stroke survivors (Chen et al., 2020; Mehrholz et al., 2018; Wu et al., 2021) with a range of devices now commercially available and being used in practice (Fasoli, 2021; Stroke Foundation, 2020). Robotics has been established as an effective intervention for improving UL strength and motor function and is recommended globally in stroke guidelines (Mehrholz et al., 2018; Morone et al., 2021; Stroke Foundation, 2024; Wu et al., 2021). However, research has primarily focussed on determining the efficacy of RT‐ULT with limited evaluation of the acceptance and usability of RT‐ULT. Insights can be found in directly exploring the perceptions and experiences of therapists.

Proctor et al. (2011) describe intervention acceptability as ‘the perception among implementation stakeholders that a given treatment, service, practice, or innovation is agreeable, palatable, or satisfactory’ (Proctor et al., 2011, p. 67). Integral to acceptance is the concept of usability, which considers the practical elements such as time and effort involved in using a device in the specific clinical context to achieve the intended therapeutic goals (International Organization for Standardization, 2018). The focus of this study was to explore the factors, which influence therapist acceptance and use of RT‐ULT in routine practice.

A number of factors have been reported that have facilitated the acceptance and use of RT‐ULT by therapists (Lo et al., 2020; Mashizume et al., 2021; Stephenson & Stephens, 2017). A strong motivator for therapists was the opportunity to provide highly intensive practice for patients with minimal input from therapy staff (Lo et al., 2020;Mashizume et al., 2021 ; Stephenson & Stephens, 2017). This was particularly appealing for stroke survivors with more severely impaired UL (Lo et al., 2020; Mashizume et al., 2021). Therapists perceived that RT‐ULT was effective for improving UL impairment and motor function for stroke survivors (Lo et al., 2020; Mashizume et al., 2021) and that these gains led to improvement in performance of activities of daily living (Mashizume et al., 2021; Stephenson & Stephens, 2017). RT‐ULT practice had the advantage of being able to be adjusted in the set‐up and training protocols to provide a ‘just right’ challenge for stroke survivors (Lo et al., 2020; Mashizume et al., 2021). Finally, RT‐ULT evaluative tools were considered helpful for objectively monitoring patient progress and detecting subtle changes in motor performance and hence facilitated acceptance of the RT‐ULT (Mashizume et al., 2021; Stephenson & Stephens, 2017).

Alternatively, therapists' have identified challenges to RT‐ULT use in practice (Celian et al., 2021; Lo et al., 2020; Stephenson & Stephens, 2017), including limited availability of staff skilled and confident in the use of RT‐ULT, time pressures associated with using a new device, the absence of published guidelines, and low numbers of suitable patients (Celian et al., 2021; Lo et al., 2020; Stephenson & Stephens, 2017). Many of these challenges are common to the use of any new intervention and likely due to limited pre‐planning and training (Lo et al., 2020; Scott et al., 2024).

These studies provide preliminary insights into the acceptance and usability of RT‐ULT but have involved small samples sizes, considered RT‐ULT only as one part of a suite of rehabilitation technology or focussed on the design process as opposed to use in routine practice (Celian et al., 2021; Lo et al., 2020; Stephenson & Stephens, 2017; van Ommeren et al., 2018). There is a need to further expand this small body of research particularly to facilitate future implementation of RT‐ULT within the Australian clinical setting.

Use of a theoretical framework can help guide the exploration of therapist perceptions of a new intervention and support the identification of targeted implementation strategies (Atkins et al., 2017). The Theoretical Domains Framework (TDF) is a validated framework that enables the analysis and categorisation of variables influencing health professionals' behaviour particularly as it relates to new or novel practices (Michie et al., 2005). The TDF has been previously used to explore therapists' perceptions of RT‐ULT in the clinical setting (Flynn et al., 2019) as well as other new forms of UL intervention for stroke survivors (Cahill et al., 2021) and national stroke guidelines (McCluskey et al., 2013). The TDF encompasses 14 domains: knowledge, skills, social/professional role and identity, beliefs about capabilities, optimism, beliefs about consequences, reinforcement, intentions, goals, memory/attention/decision process, environmental context and resources, social influences, emotion, and behavioural regulation (Atkins et al., 2017). A structured and multifaceted exploration of therapists' perceptions of RT‐ULT is important when considering the upfront expense of procuring and implementing RT‐ULT, and the crucial role therapists have in this process.

The aim of this study was to explore, through the lens of the TDF, Australian therapist acceptability and usability of RT‐ULT in their routine clinical practice.

## 2 METHODS

### 2.1 Design

A pragmatic qualitative approach was used to explore therapists' perspectives of the use of RT‐ULT in their rehabilitation facility. A pragmatic approach is characterised by a focus on understanding practice change (use of RT‐ULT in routine practice) and guidance from a framework (TDF) (Ramanadhan et al., 2021). Further to this, analysis can be strengthened in a pragmatic approach by comparing results from different methods of inquiry (Ramanadhan et al., 2021). Therefore therapists' perspectives were explored using both a focus group and individual survey, the System Usability Scale (SUS). The SUS is a brief survey that provides subjective data regarding the perceived usability and overall acceptance of a product or service (Bangor, 2009). The intention of the SUS was to help clarify and quantify individual participant perspectives of RT‐ULT and triangulate with findings from the focus groups.

This study is the fourth and final study from a series of studies that have investigated the use of RT‐ULT within this facility (Flynn et al., 2019, 2020, 2021). Ethical approval was gained from the institutions' human research ethics committee for this series of studies HREC/16/QPCH/36..

### 2.2 Site

Participants were therapists working at an inpatient rehabilitation facility located in metropolitan Queensland, Australia. The rehabilitation unit is a ‘mixed rehabilitation facility’ with patients with a variety of diagnoses participating in rehabilitation at the facility (Stroke Foundation, 2020). The facility services 42 rehabilitation beds with approximately 600 rehabilitation admissions per year, of which approximately 30% of patients have a primary diagnosis of stroke. Stroke survivors are generally seen by both occupational therapists and physiotherapists on a daily basis, Monday to Friday. There is a mix of therapy staff working either full time or part‐time.

Patient rooms and dining areas are located on the floor above the rehabilitation gym. Patients are brought to the ground floor rehabilitation gym by support staff to attend their therapy sessions. Although interconnected by a short hallway, the occupational therapy and physiotherapy areas are two distinct spaces of the rehabilitation gym. The robotic device was permanently located in the physiotherapy gym area of the rehabilitation facility.

### 2.3 Routine practice

Stroke survivors received up to 3 hours therapy per day comprised of occupational therapy, physiotherapy, and speech pathology and individualised to the person's impairments and activity limitations at this rehabilitation facility. Occupational therapists, physiotherapists, and allied health assistants were involved in providing upper limb therapy for stroke survivors at rehabilitation facility with weekly case conference. Upper limb therapy following stroke could be the focus of an entire session or provided as part of a session that included other interventions provided at this rehabilitation facility. It might be included in a physiotherapy session in conjunction with mobility and transfer retraining or in conjunction with activities daily living retraining in an occupational therapy treatment session. Upper limb therapy may be provided either one‐on‐one or in a group session at this rehabilitation facility.

### 2.4 Device

The InMotion is a robotic device that enables movement at the shoulder and elbow with an additional hand module if required. The InMotion adapts to the person's own active movements facilitating active‐assisted exercise (Bionik Labs, 2024). The device also includes a series of inbuilt evaluative tools. Users complete a pre‐programmed treatment protocol with therapy tasks presented on an adjoining computer screen. Users can also complete additional therapeutic computer games on the device (including ‘maze’, ‘pong’, and ‘squeegee’), but these games are performed using only active movements of the stroke affected upper limb (as opposed to active‐assisted of the stroke affected UL).

Although the InMotion device was permanently located in the physiotherapy gym area of the rehabilitation facility, it was anticipated that the device would be accessed by both occupational therapists and physiotherapists when the device was installed. The procurement of the device was initiated and carried out by a senior physiotherapist at the rehabilitation facility. Senior physiotherapy staff also led the training of both occupational therapists and physiotherapists in the use of the InMotion. Training sessions were ad hoc and delivered either one‐on‐one or in pairs. All new staff were also provided with one‐on‐one training as part of their orientation to the facility. Group refresher training sessions were also provided by senior physiotherapy staff.

### 2.5 Procedure

Focus group questions were informed by findings from the previous studies undertaken as part of the broader research program at the facility investigating the InMotion (Flynn et al., 2019, 2020, 2021) and through the use of the TDF. See Table 1 for a list of the semi‐structured questions used to guide the focus groups. Initial questions pertained to the introduction of the InMotion system into the participating facility rehabilitation program at both a facility and personal level and as part of routine clinical practice for stroke survivors. Participants were asked about the usefulness of the device to support semi‐supervised practice and specific aspects of the device.

**TABLE 1:** Semi‐structured interview questions.

Do you think the InMotion system has been a useful addition to the rehabilitation service—Why or why not? (Probes—impact on patient outcomes, which clients would benefit/not benefit, increasing repetitions/practice) Do you think the InMotion system has been a useful addition to your personal daily practice—Why or why not? (Probes—part of role, who uses the most, and why) Tell me about how the InMotion is used in daily practice here at (removed)? (Probes—As part of or instead of other upper limb interventions, who to use with, when to stop using) Have there been any obvious barriers to incorporating the InMotion system into your daily practice? (Probes—Sufficient skills and knowledge in use, challenges with single device, and location of device) What has helped make your use of the InMotion easier or supported your learning and practice with the device? Have management been involved directly in the implementation process? Training? Support from other staff? How do you feel patients have responded to the InMotion being part of their therapy program? (Probes—Like/do not like, pain, and negative reaction) On a really practical level, how have you found setting up the device? (Probes—Set‐up, games used, use of hand module, evaluative qualities, reliability of device, technical skills required, technical support, and safety) What would be your advice to other rehabilitation facilities who are considering purchasing and implementing an upper limb robotic device?

The TDF Version 2 (Atkins et al., 2017) was also used to inform questions and deductively analyse the transcripts. Recognising that not all domains could be sufficiently explored in the focus groups, the research team reviewed the TDF domains to prioritise five out of the 14 domains perceived to be most pertinent to therapists interviewed. The five domains prioritised were optimism, knowledge and skills, environmental context and resources, social/professional role and identity, and belief about consequences. These five domains had been discussed in depth as part of the pre‐introduction focus groups run at the facility to explore occupational therapists' and physiotherapists' pre‐conceptions of RT‐ULT (Flynn et al., 2019). For example, in the pre‐introduction focus groups the domain of social/professional role and identity was strongly represented. Therefore, in this current study, participants were asked ‘As an occupational therapist or physiotherapist, do you see the use of the InMotion device as part of your role?’

The SUS was completed individually as convenient by participants in paper form at the time of the focus groups to provide a more comprehensive picture of therapists' experiences of using the InMotion. The SUS entails 11 questions, 10 Likert scale questions, and a single overall adjective rating scale of the user‐friendliness of the product (i.e., worst imaginable, awful, poor, OK, good, excellent, and best imaginable) (Bangor, 2009; Bangor et al., 2008). A total usability score is calculated ranging from 0 to 100. Total scores can be considered in terms of the following ranges; below 50 not acceptable, 50–70 marginal, and above 70 acceptable (Bangor et al., 2008; Radder et al., 2018). The original SUS terminology of ‘system’ was used in the survey completed by participants to refer to the InMotion device as opposed to the updated SUS terminology of ‘product’ (Bangor et al., 2008). This choice of the term ‘system’ to describe the InMotion was to be consistent with the terminology used in the InMotion user manual.

Focus groups were led by one investigator (NF). This investigator had also conducted pre‐introduction focus groups (Flynn et al., 2019). Only this investigator and the participants were present at the time of the focus groups. All therapists participating in the focus groups were aware of the broader research program and had previously met the interviewer. Focus groups for this study were made up of a convenience sample of therapists who were working on the day the focus groups were held. Therapist participation was voluntary; however, all therapists available and eligible on the day consented and participated in the interviews. Focus groups were conveniently held in staff rooms adjoining the gym area, which aided recruitment and minimised the impact of the study on the therapists' daily routine. It was anticipated that focus groups would last approximately 45 minutes. Therapists who had participated in the pre‐introduction focus groups had already given their consent to participate in the post introduction focus groups. Approximately, 1 month prior to the focus groups, a senior occupational therapist distributed via email to all therapists in the department the date, time, and purpose of the focus groups as well as the participant information letter and the consent form. For those therapists who were new to the program of research, consent was gained prior to participation in the focus groups. The senior physiotherapist involved in the procurement of the device was not involved in the focus group. The two focus groups were discipline‐specific; occupational therapists and physiotherapists, enabling a clearer analysis of individual discipline perspectives on RT‐ULT, as well as providing opportunity for discussion of more discipline‐specific factors if they emerged.

Focus groups were audio recorded and transcribed verbatim for content analysis. Anticipated duration of the interviews was 45 minutes. To minimise the impact of the study on participants' transcripts were not returned. However, the investigator took notes during the course of the focus groups, which enabled an accurate summary of the key points to be communicated to and confirmed by participants at the end of each focus group. Focus group transcripts were then entered and stored in NVivo, a qualitative research software program, for analysis. Transcripts were deductively analysed with all statements in the transcripts coded against the TDF. All 14 domains were listed as potential codes, and statements could be coded to more than one domain. Two investigators (NF and DC) separately coded the transcripts using the TDF guide (Atkins et al., 2017). Despite five domains having been prioritised in the questions investigators were open to assigning relevant statements into any one of the 14 domains. Investigators then met to achieve consensus on coding and to allocate all statements under a single domain. A third investigator (SK) reviewed the coded statements to help finalise categorisation.

## 3 POSITIONALITY STATEMENT

NF is a male researcher and educator with over 20 years clinical experience as an occupational therapist and this study was conducted as part of his PhD. EF is a female professor of occupational therapy, national head of school of allied at Australian Catholic University and co‐supervisor of NF as part of his PhD. DC is a female researcher and educator with over 30 years clinical experience as an occupational therapist. She is co‐supervisor of NF as part of his PhD. SK is a female professor of physiotherapy at Australian Catholic University as well as an APA Neurological Physiotherapist. She is co‐supervisor of NF as part of his PhD.

## 4 RESULTS

### 4.1 Participants

Table 2 summarises participant demographics and System Usability Scale (SUS) scores. Two discipline‐specific focus groups were conducted with a total of nine participants (five occupational therapists; four physiotherapists). The duration of focus groups was 47 minutes with occupational therapy participants and 36 minutes with physiotherapy participants. Occupational therapy participants were predominantly female (*n* = 4), had a median age of 34 years (range 21–41 years), median of 10 years post‐qualification (range <1–20 years) with a median of 5 years' experience in working with neurological clients (range <1–15 years). Physiotherapy participants were also predominantly female (*n* = 3), had a median age of 35.5 years (range 24–54 years), median of 13.5 years post‐qualification experience (range 2–30 years), and had a median of 11 years' experience working with neurological clients (range 2–25 years). Therapists were also asked to rate their level of experience with the InMotion in terms of ‘no experience’, ‘minimal experience’, ‘experienced’, or ‘expert’. Two occupational therapists described themselves as ‘experienced’, and the remaining three described themselves as having ‘minimal experience’. One of these three occupational therapists acknowledged they had only used the InMotion as part of the training and not in practice with their own patients. Of the physiotherapists, one described themselves as ‘expert’, two ‘experienced’, and one as having ‘minimal experience’.

**TABLE 2:** Participant demographics and system usability scale (SUS) scores.

Participant	Discipline	Gender	Age range (years)	Years' experience	Years' experience in neurorehabilitation	SUS score of InMotion	SUS Q11 adjective rating of InMotion
1	OT	Female	25–30	4	4	50	Ok
2	OT	Female	21–24	0.5	0.5	60	Good
3	OT	Male	40–44	20	15	60	Good
4	OT	Female	35–39	15	10	75	Good
5	OT	Female	30–34	10	8	50	Good
6	PT	Female	50–54	30	25	85	Excellent
7	PT	Female	40–44	18	15	90	Excellent
8	PT	Male	20–24	1.5	1.5	52.5	Good
9	PT	Female	3034	9	7	70	Good

Abbreviations: OT, occupational therapy; PT, physiotherapy.

Occupational therapists' average SUS score was 59.0 (50.0–75.0) and the physiotherapists 74.0 (52.5–90.0). In terms of participants' adjective ratings of the user‐friendliness of the device, one occupational therapist assigned ‘OK’, and the remaining four occupational therapists selected ‘Good’. Two physiotherapists rated the system as ‘Excellent’ and the other two physiotherapists ‘Good’.

### 4.2 Responses

Nine out of the 14 TDF domains were discussed in depth by participants during the focus groups; environmental context and resources, beliefs about consequences, knowledge, skills, decision‐making, reinforcement, social influences, social and professional role and identity (single domain), and beliefs about capabilities. The remaining five domains were not included in the results as these categories were only discussed superficially (i.e., three comments coded to optimism) or not at all (i.e., no comments coded to emotion, goals, intentions, and optimism). Sub‐themes were created within the ‘environmental context and resources’ domain to further define specific constructs. The domains of ‘knowledge’ and ‘skills’ have been combined; however, participants' original statements were categorised under the relevant domain during analysis.

#### 4.2.1 Environmental context and resources

Participants identified a number of environmental and organisational factors that they believed influenced the use of the RT‐ULT device.

##### Physical location

The primary environmental influence on the use of the InMotion in daily practice reported by therapists was the physical location of the device in the physiotherapy area of the gym. Physiotherapists highlighted how this was particularly conducive to their practice.

It’s in the middle of the gym … you’re always reminded that its around and thinking who you could use it with. (Physiotherapist 3)

Being accessible and in the gym, you can set people up and still work with other patients rather than having to be in a separate area or separate room. (Physiotherapist 4)

Alternatively, the occupational therapists identified that the location of the device did mean they did not use the device as often as their physiotherapy colleagues.

It is in the far end (of the gym), it’s almost out of sight out of mind for me sometimes. (Occupational Therapist 3)

I mean part of its environmental here, we’ve got two very separate spaces … It’s probably in the best space to get the most use. (Occupational Therapist 1)

In some way perhaps the way we work as OTs is part of that … we don’t tend to have the same continuous cycle through the gym space … you’re often upstairs dealing with other ADL (Activities of Daily Living) type practice or something like that which we can’t do in a gym space. (Occupational Therapist 1)

Set‐up of the device. Perceptions were mixed when participants reflected on the setting‐up of the device.

Certainly doesn’t take half an hour. It’s one of the advantages of it is that it is quite quick. (Physiotherapist 3)

Some people who are, just really don’t have the endurance … to kind of perform it for a feasible amount of time. It’s not really worth the set up. (Physiotherapist 2)

##### Organisational challenges

Participants described a range of organisational factors that impacted their use of the device, including personal work schedules and admission rates of suitable patients for its use.

And it’s tricky because I'm only here part‐time and haven’t been here that long and probably haven’t a huge number of patients that the robot’s been suitable. (Occupational Therapist 4)

It probably depends on the mix of patients that we’ve got at that time. Earlier in the year we were through summer really busy with stroke patients, so it was used quite a lot. (Physiotherapist 3)

In terms of actually embedding it into our practice I don’t feel like we ever had the luxury of focusing on that in a really structured way. There’s been like ad hoc kind of attempts. (Occupational Therapist 2)

#### 4.2.2 Beliefs about consequences

Overall, both disciplines were very positive with regard to the perceived clinical advantages of having the InMotion part of routine practice.

It definitely provides a very high intensity and repetition count for patients especially with densely affected upper limbs. (Physiotherapist 1)

It’s definitely been a change for us in terms of getting overall dosage. (Occupational Therapist 2)

You don’t have to stress about being there one on one with them. The machine really is that one assist that they need which is really cool. (Physiotherapist 2)

Gives them some variety in their treatment as well so it’s not so mundane day in day out. (Physiotherapist 2)

Participants did stress that RT‐ULT was just one part of their overall approach to facilitating UL recovery with their patients.

My biggest piece of advice would be that it’s not the answer. It’s a part of the therapy that we offer. Yes, I think overall it’s definitely beneficial, but it shouldn’t replace upper limb therapy. (Physiotherapist 1)

There’re very few patients that would just do the robot. They’re normally having a combination of evidence‐based interventions. (Physiotherapist 3)

I think it’s been a useful adjunct to the other functional things that we would typically do … I think it’s been useful from a team perspective. (Occupational Therapist 1)

#### 4.2.3 Knowledge and skills

Participants provided reflection in relation to their personal knowledge and skill in using the device. Physiotherapists, as the main prescribers of RT‐ULT, reported a familiarity with using the pre‐programmed treatment protocols of the InMotion but acknowledged a need to understand more of the other functions.

I feel like I know the real basics to it, the core fundamentals but I'm not sure how I’d go with explaining all of it, the real intricacy. (Physiotherapist 2)

If you are going into the nitty gritty the robotic reports and things, I think they’re quite hard to understand. (Physiotherapist 1)

The physiotherapists also described how it had been a relatively easy process to acquire their knowledge and skill in the use of the device.

You can kind of teach yourself once you know the basics you can just sit down in a spare half an hour and just have a play on it, figure out how it works. (Physiotherapist 1)

It probably depends on your own learning style but I often find its easier for me to get on and do it myself. (Physiotherapist 3)

Alternatively, the occupational therapists described a limited knowledge and skill base.

I haven’t had a lot of patients so it’s difficult to develop your skills and confidence using it when it’s so sporadic. (Occupational Therapist 1)

I think a more thorough orientation may have encouraged me to use it more or have a bit more confidence to identify “I think that patient would benefit”. (Occupational Therapist 4)

I can remember parts of it, but I still wouldn’t be able to go and take a patient through it now since I haven’t used it since receiving that information (training). (Occupational Therapist 5)

Both therapy disciplines reflected on the challenges in using the optional hand module of the InMotion.

But the hand piece was a little bit confusing. (Occupational Therapist 3)

The hand piece can be a little bit technical to put on. (Physiotherapist 1)

A lack of knowledge from referral sources was highlighted as influencing the use of the InMotion.

I think there was a little bit of misconception by others (referring agencies) out there as well. That they were hearing about this magical robot thing. And like we were getting referrals from other rehab units saying, referring people just for the robot and they didn’t really understand like how it was being utilised … It wasn’t like it was going to be the cure. Which is kind of what the impression was. (Occupational Therapist 1)

#### 4.2.4 Decision processes

Participants provided key insight into their reasons for deciding when and why to incorporate RT‐ULT into their patient rehabilitation programs.

I think it’s when we know someone’s come in with a dense hemiplegia, we prioritise trying to get them started. (Physiotherapist 3)

I would tend to identify it as an initial modality. (Occupational Therapist 2)

98% of the time I’m only using the robotic program (active‐assisted movements) itself rather than the other 5 options (active movements only) … instead of using them I would use more functional based activities … So mainly I use the robot for the active‐assisted therapy it offers. (Physiotherapist 1)

Physiotherapists also highlighted where they felt RT‐ULT was not indicated for their patients.

To train that grasp I’d probably preference other treatments. (Physiotherapist 2)

The other games I don’t find that helpful in terms of training the patient in a functional way. If they’re at the skill level where they can actively move the robot … . I would start doing more functional based tasks with them. (Physiotherapist 1)

Physiotherapists reported using the hand module on occasions when the patient had good distal return of movement but identified the limitations of the hand module.

I’ve used it with a couple of patients that have more distal weakness in their stroke… was a nice component of their program. (Physiotherapist 3)

Tryin’ to train that grasp I’d probably preference other treatments over just that open, close, open, close, open, close. (Physiotherapist 2)

It’s not really a functional grasp it’s just a lumbricals’ exercise really. (Physiotherapist 1)

It was also flagged by one of the occupational therapists how the evaluative elements of the InMotion can make clear where patient movement patterns are breaking down and, in doing so, assist in treatment planning.

The reports are quite interesting for isolating the type of movement they’re getting or the area where their deficit is … when I have taken time to look at the data, I do find that it tends to make me go back to the drawing board maybe with the exercises I am prescribing away from the robot. (Occupational Therapist 2)

#### 4.2.5 Social and professional role and identity

Both therapy disciplines recognised that the physiotherapists had taken the lead with the use of RT‐ULT in routine practice but neither discipline perceived this to be a negative aspect.

I don’t see it’s a massive problem that the physios are taking the primary lead … I think when it comes down to it, is the patient using it? Is the patient benefiting from it? Regardless of who’s actually doing that. (Occupational Therapist 1)

I mean it’s often the physios that are starting off on the InMotion but it doesn’t have to be. (Physiotherapist 3)

Culturally there’s a lot of blurring between the disciplines so I think it’s sort of just negotiated generally rather than it being actually recorded or directed to anywhere in particular. (Occupational Therapist 2)

It was also an expectation that students would be able to incorporate RT‐ULT into their patient rehabilitation programs.

Certainly, the ones (students) on clinical placement if their patients are indicated for it then yep, it’s an expectation that they’ll be confident enough, or you know train them up to be confident enough to set patients up on it and it’s just part of their therapy. (Physiotherapist 3)

#### 4.2.6 Reinforcement

Therapists described how patients' use of RT‐ULT was reinforced by the visual feedback provided on the computer screen of the InMotion and the ability to independently operate the machine.

Surprised me with how interested they are. All of the games are quite simplistic. (Occupational Therapist 2)

They do tend to fixate on that robot screen really well especially patients who are easily distracted they seem to respond really well to the screen of the robot. I’ve always been kind of like pretty amazed at how attentive they can be to a robot screen when they’re otherwise quite distractible. (Physiotherapist 1)

The feedback that I’ve been consistently getting from them is that they feel like they can do things on it that they can’t trying to complete other activities or exercises. (Occupational Therapist 4)

I think the patients really enjoy the fact that they can operate it themselves once they’ve learnt how to do it. That’s one thing I’ve noticed they’re you know really quite engaged and it’s like they’re driving their own therapy, which I think is really important. (Physiotherapist 3)

#### 4.2.7 Social influence

Broader influences, including patients' family and management at the facility, were believed to have enhanced the use of the InMotion device in practice.

I also find families really like it as well when they’re coming down to see the patient strapped to a fancy schmancy, you know, very expensive piece of technology they, families do tend to enjoy that. (Physiotherapist 1)

And it’s a cool point of difference as well. Because seems to be like when execs (executives) come through they’re always looking at the robot. And it’s just I guess one of those things that sets us aside. (Physiotherapist 2)

#### 4.2.8 Beliefs about capabilities

Therapists acknowledged that their own level of confidence certainly plays a part in initial use of the device.

Although I think its reasonably easy to use. I just don’t have the confidence because it’s been so erratic when it’s been relevant for me to use. (Occupational Therapist 4)

It’s the confidence initially. Whereas now I know how to use it quite well it’s not really an issue anymore. (Physiotherapist 2)

## 5 DISCUSSION

Much of RT‐ULT research to date has focussed on determining the efficacy of this form of treatment (Mehrholz et al., 2018; Wu et al., 2021), but these trials provide limited insight into the factors influencing the acceptance and use of RT‐ULT into routine clinical practice. This study used the TDF to categorise factors influencing therapist acceptance and use of RT‐ULT as part of routine practice. Nine out of the 14 TDF domains were discussed in depth during focus group discussions with occupational therapists and physiotherapists. To the authors' knowledge, this is the first program of research to apply behavioural theory to explain the factors influencing therapists' acceptance and use of RT‐ULT when part of routine practice. This is important as successful implementation is principally dependent on behavioural change of health professionals (Atkins et al., 2017). This study was also informed by findings from three previous studies which were part of a broader research program exploring the introduction of RT‐ULT at the rehabilitation facility.

The responses of therapists in this study indicated that they were generally accepting of the introduction of RT‐ULT into routine clinical practice, particularly for stroke survivors. The acceptance of a new intervention can be linked to the therapists' overall perception that the advantages outweigh the disadvantages (Wensing et al., 2020). Therapists were able to clearly identify the clinical advantages of RT‐ULT in terms of the capacity to increase the amount and intensity of practice for patients, facilitate semi‐supervised, or independent practice and providing a motivating form of upper limb task practice.

RT‐ULT was perceived to be particularly relevant for stroke survivors with dense UL hemiplegia. The clinical relevance and advantage of RT‐ULT for this group of stroke survivors was also highlighted previously (Lo et al., 2020) in a study exploring therapist perceptions of upper and lower limb robotics. Clinical trials indicate that the use of RT‐ULT can address UL impairment with stroke survivors who have moderate to severe UL deficits (Hesse et al., 2014; Klamroth‐Marganska et al., 2014; Rodgers et al., 2019). However, RT‐ULT has not consistently been found to be more effective than usual therapy on outcome measures evaluating activity and participation (Chen et al., 2020; Ferreira et al., 2021; Mehrholz et al., 2018; Veerbeek et al., 2017). It was reported in this current study that once stroke survivors demonstrated sufficient return of active UL movement, then functional‐based UL practice took priority and RT‐ULT was less utilised or ceased. These perceived limitations of RT‐ULT may explain why the domain of optimism did not emerge as a clear theme. Optimism refers to the confidence a therapist has that a desired goal will be achieved with the use of a new intervention (Atkins et al., 2017). Therapists in this study recognised that in order for patients to achieve their recovery goals that a combination of UL interventions needed to be incorporated not just RT‐ULT alone.

Although it was reported by both occupational therapists and physiotherapists in the focus groups that the introduction of RT‐ULT had been a positive addition to the rehabilitation facility, it was the physiotherapists who were the primary prescribers of the InMotion device. This had been previously established in a conjoint study (Flynn et al., 2021), which investigated the sustainability of RT‐ULT in routine practice at the facility, and which found that 80% of RT‐ULT sessions in the initial 2 years following its introduction had been prescribed by the physiotherapists. The primary reason for physiotherapists in this clinical setting being the primary prescriber of RT‐ULT appeared to be that the InMotion was located in the physiotherapy area of the gym. The physical location of a new intervention can influence the uptake in routine practice, with availability at the point of care being important for uptake. Lo et al. (2020) identified that the location of a robotic device was an important consideration often overlooked when introducing the device. In the focus group with the occupational therapists, it was identified that the location of the InMotion in the physiotherapy area meant they had been less inclined to incorporate RT‐ULT into their practice. The occupational therapists also highlighted that other clinical priorities (i.e., functional retraining on the ward) and staff employment arrangements (i.e., working part‐time rather than full time) had contributed to their more limited use of the InMotion. This difference in RT‐ULT use between the two therapy disciplines was not perceived by therapists as a negative outcome. One of the occupational therapists reflected that the introduction of RT‐ULT had been useful from a whole of ‘team perspective’, appreciating that UL rehabilitation was an area in which the two disciplines collaboratively worked together.

The variance in RT‐ULT use by the two disciplines was evident in their SUS ratings, with physiotherapists recording notably higher scores, reflective of their greater amount of exposure to the device. The SUS has been previously used to determine stroke survivors' perspectives of RT‐ULT usability (Nijenhuis et al., 2015; Radder et al., 2018) but not therapists' perspectives. This scale has however been used to measure health professionals' perspectives of the usability of other forms of technology such as electronic health records and 3D mapping applications for home modification design (Bloom et al., 2021; Hamm et al., 2019). Therapist's comfort with accessing and interacting with RT‐ULT technology was important to quantify in this study to compliment and confirm the perspectives communicated in the focus groups. The use of tools like the SUS in future implementation studies may help strengthen the qualitative findings of focus groups or interviews particularly when participant numbers are small. It may also be a helpful tool as part of clinical practice to gain a quick snapshot of clinician confidence in using RT‐ULT particularly if there has been an extended time period since initial training. Lower scores could be used as an indicator for staff to undergo refresher training in the use of the device.

It is somewhat remarkable how accepting therapists were of RT‐ULT in this current study when considering the ‘ad hoc’ training and support provided. Successful uptake of a new intervention, such as RT‐ULT, should ideally involve careful forward planning and strategic coordination (Lo et al., 2020). Without such planning and strategy, change in practice is likely to be minimal or short‐lived (McCluskey & O'Connor, 2017). Although not directly discussed in the focus groups, this relative success could be attributed in part to the acquisition and introduction of RT‐ULT being both initiated and led by clinicians, that is, senior physiotherapists. The importance of nominating clinical champions is a well‐established implementation strategy (Miech et al., 2018). However, what is less evident is the potential advantage and effectiveness of an implementation process that is also initiated by clinicians. The value of an implementation process that is clinician‐initiated, not just clinician‐led, merits further exploration. The fact that the acquisition and introduction of RT‐ULT was initiated and led by senior physiotherapists also likely contributed to the physiotherapists being the primary prescribers of the device.

The InMotion was described by therapist participants as being easy to use. The physiotherapists explained that once having had the initial training they were confident to independently sit down and explore the functionality of the InMotion. It was also an expectation that physiotherapy students would be able to incorporate RT‐ULT into their programs. Although the occupational therapists were more limited in their use of RT‐ULT they also described the device as being relatively easy to use. How easy a particular technology is to use is crucial to uptake. In a survey of 292 healthcare professionals, Hughes et al. (2014) identified that ease of set‐up and use was ranked the second most important quality of rehabilitation technology behind supporting evidence. One participant in this current study reported that for set‐up to be warranted a patient needed to be able to participate in RT‐ULT for a good amount of time. Also, the hand module was described as being difficult to set up with patients. Difficulty with the hand module was indicated in one of the preceding studies at the facility investigating RT‐ULT sustainability (Flynn et al., 2021), which revealed that the hand module was used only a quarter of the time by patients.

Patient acceptance of an intervention can strongly influence the use of new technology for therapists (Chen & Bode, 2011). Therapist participants in this study perceived that RT‐ULT was well‐received by patients and their families. Participants reported that patients enjoyed being able to practice independently on the robot, giving them a greater sense of control over their own therapy. It was also believed that patients were motivated by having greater freedom of movement when practicing on the device than compared other forms of UL therapy. Finally, the RT‐ULT games presented on the screen were observed and reported by therapists to be very engaging for patients.

## 6 STUDY LIMITATIONS

This study explored the views of a small sample of occupational therapists and physiotherapists within a single rehabilitation facility in Australia. The small sample of participants interviewed means that data saturation was unlikely to have been reached. Conclusions from this study are therefore indicative of the participants and their clinical setting and should be considered with this in mind. However, the facility is largely reflective of Australian rehabilitation services, which are typically public facilities, was located in a metropolitan area and providing mixed rehabilitation services 5 days per week inclusive of occupational therapy and physiotherapy (Stroke Foundation, 2020).

The use of the TDF to guide the development of the focus group questions and analysis process may have limited identification of other relevant themes. However, this framework facilitated a structured process of categorisation in line with other studies that have explored therapists' perceptions of a new intervention or guideline. It is also acknowledged that the inclusion of patient participants alongside therapists would have provided a more comprehensive picture of the implementation process. It is possible that prior exposure of the participants to the interviewer (e.g., knowing what they were interested in and knowing what they valued) might have cued participants to certain response types or views.

## 7 CONCLUSION

RT‐ULT is recommended in international guidelines for stroke survivors, but there is limited understanding of the acceptability and use of RT‐ULT by therapists in routine practice. This study explored occupational therapists and physiotherapists perception of RT‐ULT 20 months following its introduction. RT‐ULT was generally perceived by therapists to be an acceptable form of therapy for stroke survivors, enabling an increase in the number of repetitions of upper limb movement practice and intensity of independent practice. The use of RT‐ULT in routine practice was influenced by the physical location of device, simple functionality, and strong patient acceptance. Future research should investigate locating the device in the occupational therapy area of the facility to determine if this impacts their use of the device. Additionally, the potential advantages of clinician‐initiated implementation of practice innovations should be investigated as well as exploration of patient stroke survivors' perceptions of RT‐ULT use as part of their rehabilitation programs.

## AUTHOR CONTRIBUTIONS

All authors contributed to the design of this research. Nicholas Flynn completed data collection. Nicholas Flynn, Deirdre Cooke and Suzanne Kuys undertook data analysis. All authors contributed to the write up of the manuscript.

## CONFLICT OF INTEREST STATEMENT

All authors declare that there were no conflicts of interest in this study.

## Data Availability

The data that support the findings of this study are available from the corresponding author upon reasonable request.
